# Isolation and Characterization of an LBD Transcription Factor CsLBD39 from Tea Plant (*Camellia sinensis*) and Its Roles in Modulating Nitrate Content by Regulating Nitrate-Metabolism-Related Genes

**DOI:** 10.3390/ijms23169294

**Published:** 2022-08-18

**Authors:** Rui-Min Teng, Ni Yang, Jing-Wen Li, Chun-Fang Liu, Yi Chen, Tong Li, Ya-Hui Wang, Ai-Sheng Xiong, Jing Zhuang

**Affiliations:** 1 Tea Science Research Institute, College of Horticulture, Nanjing Agricultural University, Nanjing 210095, China; 2 State Key Laboratory of Crop Genetics and Germplasm Enhancement, Nanjing Agricultural University, Nanjing 210095, China

**Keywords:** tea plant, nitrate, expression level, transcription factor, overexpression

## Abstract

Nitrate nitrogen is an important nitrogen source for tea plants’ growth and development. LBD transcription factors play important roles in response to the presence of nitrate in plants. The functional study of LBD transcription factors in tea plants remains limited. In this study, the LBD family gene *CsLBD39* was isolated and characterized from tea plants. Sequence analysis indicated that CsLBD39 contained a highly conserved CX_2_CX_6_CX_3_CX domain. The phylogenetic tree assay showed that CsLBD39 belonged to class II subfamily of the LBD family. *CsLBD39* was highly expressed in flowers and root; we determined that its expression could be induced by nitrate treatment. The CsLBD39 protein was located in the nucleus and has transcriptional activation activity in yeast. Compared with the wild type, overexpression of *CsLBD39* gene in *Arabidopsis* resulted in smaller rosettes, shorter main roots, reduced lateral roots and lower plant weights. The nitrate content and the expression levels of genes related to nitrate transport and regulation were decreased in transgenic *Arabidopsis* hosting *CsLBD39* gene. Compared with the wild type, *CsLBD39* overexpression in transgenic *Arabidopsis* had smaller cell structure of leaves, shorter diameter of stem cross section, and slender and compact cell of stem longitudinal section. Under KNO_3_ treatment, the contents of nitrate, anthocyanins, and chlorophyll in leaves, and the content of nitrate in roots of *Arabidopsis* overexpressing *CsLBD39* were reduced, the expression levels of nitrate transport and regulation related genes were decreased. The results revealed that CsLBD39 may be involved in nitrate signal transduction in tea plants as a negative regulator and laid the groundwork for future studies into the mechanism of nitrate response.

## 1. Introduction

Nitrogen (N) is an important macronutrient necessary for the normal growth and development of higher plants [1,2]. For most plants, nitrate (NO_3_^−^) is the primary source of nitrogen and can be assimilated to nitrite, ammonium and amino acids [3]. Nitrate serves as an essential nutrient and an important signaling molecule [4]. Nitrate is the most established and probably the dominant nitrogen signal that modulates root architecture, leaf development, and anthocyanin accumulation [5,6,7]. The *nitrate transport* (*NRT*) gene families, nitrate reductase (*NIA*) and nitrite reductase (*NIR*) genes are involved in nitrate transport and assimilation [8,9]. Some studies have demonstrated that NLP, LBD, NRG, and other transcription factors play vital roles in regulating nitrate metabolism [1,10,11].

LBD (Lateral organ boundaries domain) gene family is one of the plant-specific transcription factor (TF) families. LBD TFs have a highly conserved LOB (Lateral organ boundaries) domain with about 100 amino acids and function as a regulation factor to modulate plant development and metabolic processes in plants [7,12]. The LBD proteins are divided into classes I and II [13,14]. Class I proteins contain a fully conserved CX_2_CX_6_CX_3_C region (zinc finger domain), a glycine-alanine-serine region (GAS) and a LX_6_LX_3_LX_6_L region (leucine zipper motif); while the class II proteins contain only one zinc finger domain [12]. Zinc finger domain was thought to be necessary for DNA binding, the GAS region can assist the binding of the CX_2_CX_6_CX_3_C region to the promoter, and the leucine zipper sequence may be involved in protein dimerization [15].

Several studies have revealed that AtLBD37/38/39 are involved in the regulation of plant nitrate metabolism and anthocyanin metabolism pathway as negative regulators [11]. MdLBD13 is a nitrate signaling factor that regulates nitrate uptake/assimilation and anthocyanin biosynthesis in apple [16]. Ectopic expression of *OsLBD37* in *Arabidopsis* interferes with nitrogen metabolism, resulting in early flowering, yellow leaves and senescence [17]. Konishi and his colleagues testified that NIN-LIKE PROTEIN (NLPs) are DNA-binding proteins that bind to the nitrate-responsive *cis*-element (NRE) region of nitrate-responsive genes. NLPs also have effects on the expression of the nitrate-inducible regulatory factor gene *LBD39* [18]. AtLBD18 binds directly to the *AtEXPANSIN14* promoter and promotes the occurrence of lateral roots in *Arabidopsis* [19]. Similarly, AtLBD18 also promotes the formation of lateral root primordia in *Arabidopsis* by regulating *AtEXPA17* gene [20]. Although there are many studies of *LBD* genes in other species, little is known about the function of *LBD* genes in tea plants.

Tea plant (*Camellia sinensis* (L.) O. Kuntze) originated in southwest of China and is now widely cultivated as a cash crop in the world [21,22]. As an evergreen leaf beverage plant, tea contains many beneficial components and is very popular among people [23]. In the process of growth and development, tea plants are vulnerable to the stress of element deficiency, resulting in the decline of tea yield and quality [24]. The nitrogen forms absorbed and utilized by tea plant are mainly ammonium (NH_4_^+^) and nitrate (NO_3_^−^). As an ammonium-loving plant, the uptake of NH_4_^+^ by tea roots was significantly higher than that of NO_3_^−^ [25]. At present, the research on nitrogen absorption and utilization mechanism of tea plant mainly focuses on NH_4_^+^. The reports on the molecular mechanism of NO_3_^−^ uptake and utilization are limited to cloning of a few nitrate transport genes [26]. Studies on the regulatory genes in tea plant have not been reported. In-depth analysis of the regulation mechanism of NO_3_^−^ absorption by tea plants is of great significance for the genetic improvement of tea plants with high nitrogen efficiency.

In this study, an LBD gene was cloned from tea plant ‘Longjing 43’ and named *CsLBD39*. The expression of *CsLBD39* is induced by nitrate treatment. The analysis of subcellular localization, transcriptional activation activity and *CsLBD39*-overexpression in *Arabidopsis* further validated the function of *CsLBD39* gene. The results of this work provided foundational knowledge for comprehending the structure and function of CsLBD39, as well as the regulation of nitrate metabolism.

## 2. Results

### 2.1. Sequence and Phylogenetic Tree Analysis of the CsLBD39

**Figure 1:**
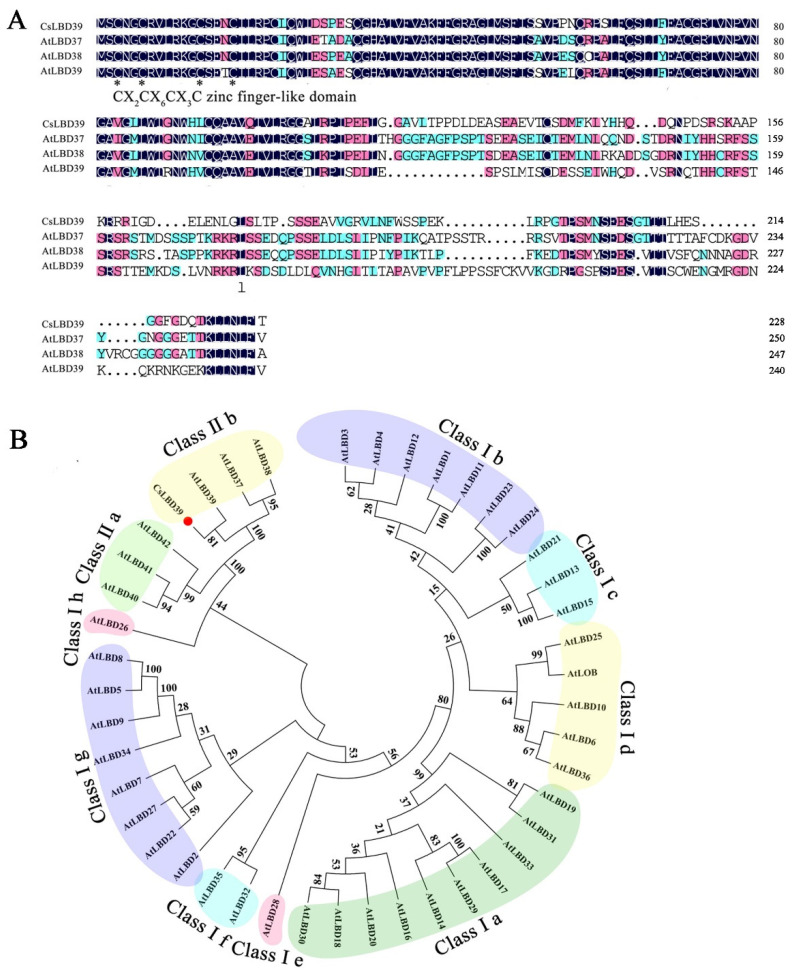
Bioinformatics analysis of the CsLBD39 protein sequence. (**A**) Multiple sequence alignments among CsLBD39 and other LBDs from *Arabidopsis*. The conserved DNA-binding domain is indicated by black asterisk. (**B**) Phylogenetic tree of CsLBD39 and AtLBDs from *Arabidopsis*. Red circle represented CsLBD39.

*CsLBD39* was isolated from tea plant ‘Longjing 43’. Sequence analysis showed that *CsLBD39* gene was 687 bp in length and encoded 228 amino acids. Multiple sequence alignments showed that CsLBD39 and other LBDs had a typical zinc finger domain (CX_2_ CX_6_CX_3_CX) (Figure 1A). In order to understand the classification of CsLBD39, the sequence of CsLBD39 and the LBDs of *Arabidopsis* were used to construct a phylogenetic tree. The results showed that CsLBD39 belongs to the class II subfamily (Figure 1B).

### 2.2. Relative Expression Level of CsLBD39 in Tea Plant

**Figure 2:**
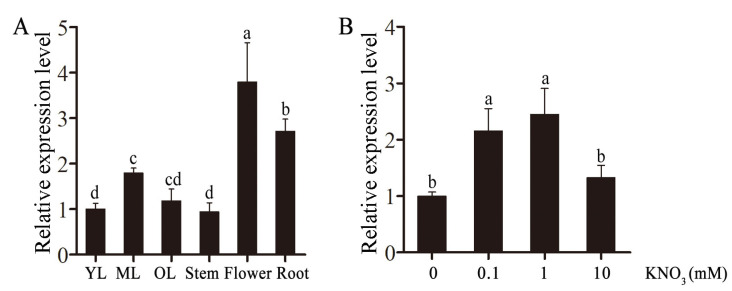
The relative expression of *CsLBD39* in tea plant. (**A**) The relative expression levels of *CsLBD39* in different developmental stages and tissues. (**B**) The relative expression levels of *CsLBD39* in the root after adding 0.1, 1, 10 mM KNO_3_ to N-limited tea plant seedlings. The data are expressed as mean ± standard deviation of three replicates (*n* = 3). Different lowercase letters indicate significant differences at *p* < 0.05.

The transcript levels of *CsLBD39* in different developmental stages and tissues were determined. The results showed that *CsLBD39* gene was expressed in all tested tissues, and the expression levels were higher in flowers and roots (Figure 2A). *CsLBD39* gene was affected by different concentrations of nitrate, and its expression reached the maximum at 1 mM KNO_3_ treatment (Figure 2B).

### 2.3. Subcellular Localization and Transcriptional Activation Activity Analysis of CsLBD39

**Figure 3:**
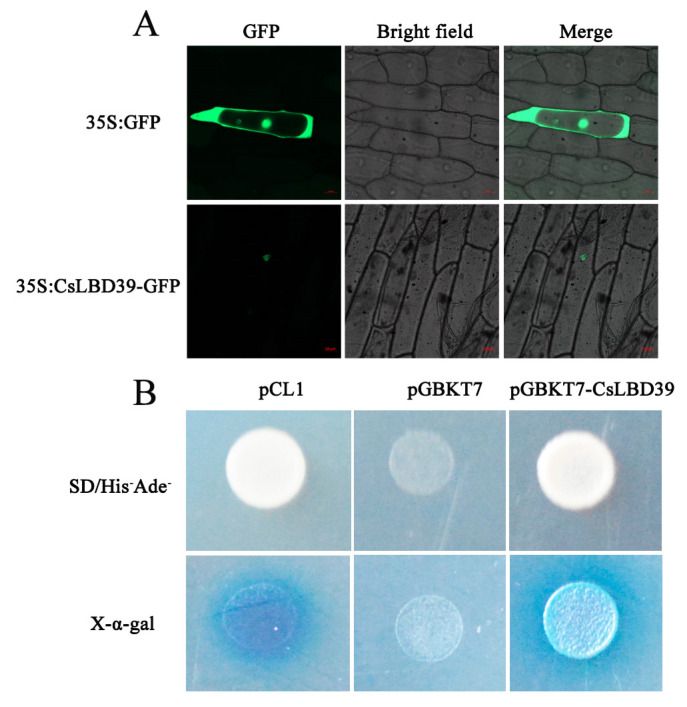
Subcellular localization and transcriptional activation activity of CsLBD39. (**A**) Subcellular localization of CsLBD39 in onion epidermal cells. Scale bars = 50 μm. (**B**) Transcriptional activation activity of CsLBD39 in yeast.

Studying where a protein is expressed is essential to determine its function [27]. CLBD39 was fused with GFP to construct recombinant vector CsLBD39-GFP. The recombinant plasmid, CsLBD39-GFP, was bombarded into onion epidermal cells using the gene gun to observe the subcellular localization. The result found that pA7-GFP fluorescence signal permeated the onion cell, and CsLBD39-GFP fusion protein is expressed in the nucleus (Figure 3A).

To detect the transcriptional activation activity of CsLBD39, CsLBD39 was constructed into pGBKT7 vector containing GAL4-binding domain to obtain the yeast expression vector pGBKT7-CsLBD39. Positive control (pCL1), negative plasmid (pGBKT7), and pGBKT7-CsLBD39 were transferred into Y2H yeast receptor cells, respectively. The yeast strains transformed with pCL1 were cultured on SD/Leu^−^ solid medium, the other two were cultured on SD/Trp^−^ solid medium, respectively. The positive yeast screened by SD/Leu^−^ and SD/Trp^−^ were inoculated on SD/His^−^Ade^−^ deficient medium with or without X-α-Gal, respectively. The results showed that pCL1 and pGBKT7-CsLBD39 could grow on SD/His^−^Ade^−^ +X-α-Gal solid medium and showed blue color, while pGBKT7 could not grow on SD/His^−^Ade^−^ +X-α-Gal solid medium, indicating that CsLBD39 had transcriptional activation activity in yeast (Figure 3B).

### 2.4. Overexpression of CsLBD39 in Arabidopsis

The *CsLBD39* gene was inserted into pCAMBIA1301 vector carrying β-glucuronidase (*GUS*) reporter gene, allowing that *CsLBD39* and *GUS* separately driven by CaMV 35S promoter, so that *CsLBD39* and *GUS* could co-express in transgenic plants (Supplementary Figure S1A). GUS staining were performed for the identification of transgenic *Arabidopsis*, finding that the cotyledons and roots of 7-day-old *Arabidopsis* showed blue color (Supplementary Figure S1B). *GUS* gene was expressed in filaments, anthers, stigmas, sepals and siliques of *Arabidopsis* (Supplementary Figure S1D). The cDNAs of WT and transgenic *Arabidopsis* were amplified by PCR to further identify the expression of *CsLBD39* in transgenic plants, showing that the corresponding bands could be detected in the transgenic lines (Supplementary Figure S1C). Then, the RT-qPCR assay also indicated that *CsLBD39* was overexpressed in transgenic *Arabidopsis* plants (Supplementary Figure S1E).

### 2.5. Changes in Fresh Weight and Roots of Transgenic Arabidopsis Overexpressing CsLBD39

**Figure 4:**
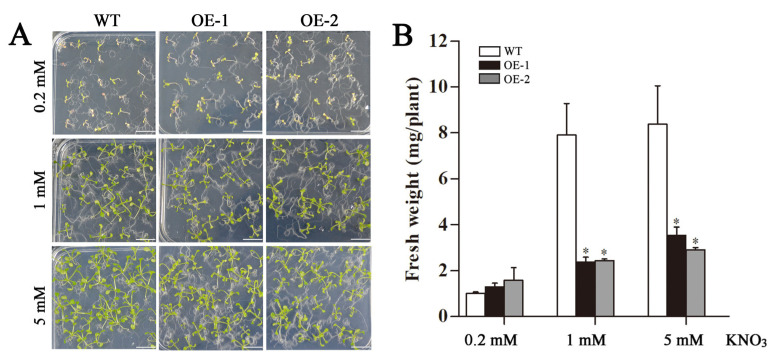
Analysis of fresh weight in WT and transgenic *Arabidopsis* hosting *CsLBD39* gene under KNO_3_ treatment. (**A**) The phenotypes of the 15-day-old plants under different KNO_3_ conditions. Bar = 1 cm. (**B**) Fresh weight of the 15-day-old plants under different KNO_3_ conditions. The data are expressed as mean ± standard deviation of three biological replicates (*n* = 3). Asterisks (*) indicate that the value is significant difference compared to the WT (* *p* < 0.05).

**Figure 5:**
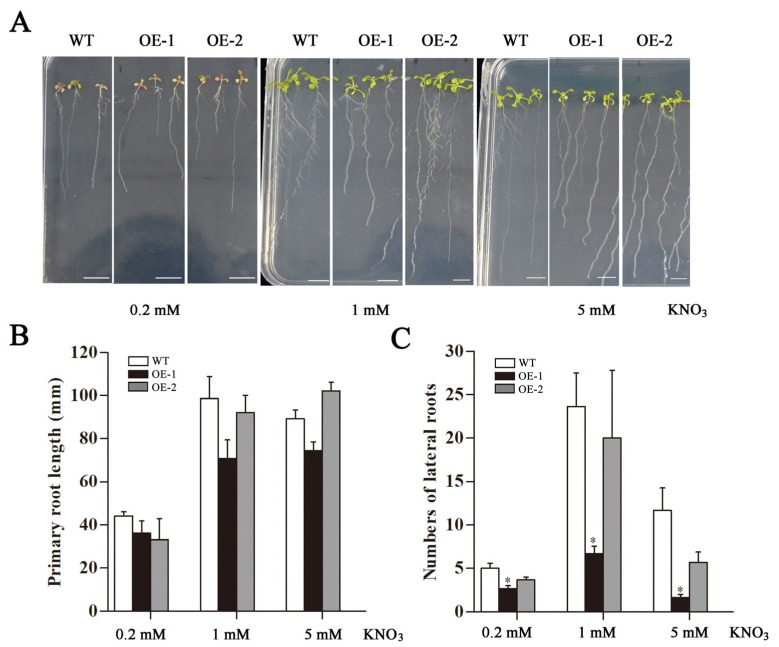
Analysis of roots in WT and transgenic *Arabidopsis* hosting *CsLBD39* gene under KNO_3_ treatment. (**A**) The phenotypes of the 15-day-old plants on vertical plates containing different concentrations of KNO_3_. Bar = 1 cm. (**B**) The primary root length (**C**) and numbers of lateral roots of the plants under different KNO_3_ conditions. The data are expressed as mean ± standard deviation of three biological replicates (*n* = 3). Asterisks (*) indicate that the value is significant difference compared to the WT (* *p* < 0.05).

Transgenic *Arabidopsis* was cultured in MS medium with different concentrations (0.2, 1, and 5 mM) of KNO_3_ for 15 days (Figure 4A). The fresh weight of transgenic lines was significantly lower than that of wild type (WT) at 1 mM and 5 mM KNO_3_ treatments (Figure 4B). The morphology of taproots and lateral roots of *Arabidopsis* were observed after 15 d (Figure 5A). The results showed that under 0.2 mM and 1 mM KNO_3_ treatments, the transgenic taproots were shorter than the WT (Figure 5B). Under the treatment of KNO_3_ at three concentrations, the number of lateral roots of transgenic *Arabidopsis* was less than that of the WT, especially the number of lateral roots of OE-1 was significantly lower than that of the WT (Figure 5C).

### 2.6. Analysis of Nitrate, Anthocyanin and Chlorophyll Contents in Transgenic Arabidopsis Overexpressing CsLBD39 Gene

**Figure 6:**
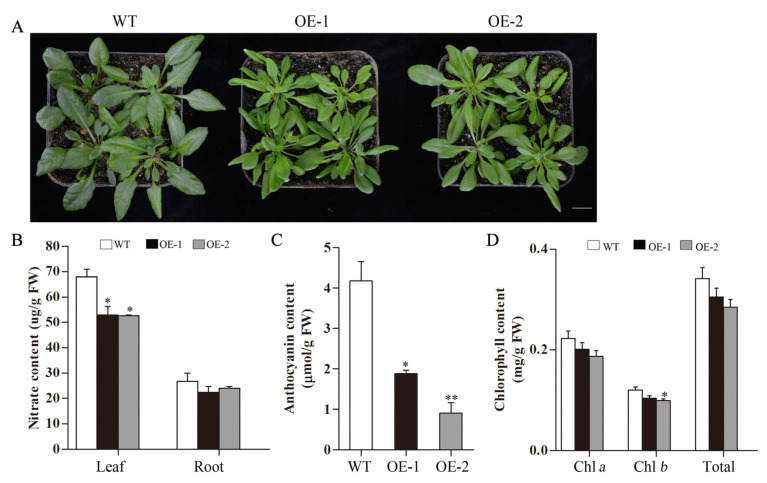
Phenotype and nitrate, anthocyanins, chlorophyll contents of transgenic *Arabidopsis* plant hosting *CsLBD39* gene. (**A**) The phenotypes of the 35-day-old transgenic *Arabidopsis* and WT plants. Bar = 1 cm. (**B**) Nitrate, (**C**) total anthocyanins, and (**D**) chlorophyll contents of 35-day-old transgenic *Arabidopsis* and WT plants. The data are expressed as mean ± standard deviation of three biological replicates (*n* = 3). Asterisks (*) indicate that the value is significant difference compared to the WT (* *p* < 0.05; ** *p* < 0.01).

35-day-old *Arabidopsis* plant was used to test the nitrate content (Figure 6A). The nitrate content in the leaves and roots of the transgenic *Arabidopsis* was lower than that of the WT, especially in leaves (Figure 6B). The content of anthocyanins was affected by nitrogen stress in plants. Here, determined total anthocyanins content in transgenic *Arabidopsis* leaves was significantly reduced (Figure 6C). We observed that the leaves of transgenic *Arabidopsis* were light green and those of the WT was dark green (Figure 6A). The content of chlorophyll *a* and chlorophyll *b* in transgenic *Arabidopsis* were reduced compared to the WT *Arabidopsis* (Figure 6D).

### 2.7. Expression Analysis of Nitrate Uptake and Transport-Related Genes in Transgenic Arabidopsis Plants Overexpressing CsLBD39 Gene

**Figure 7:**
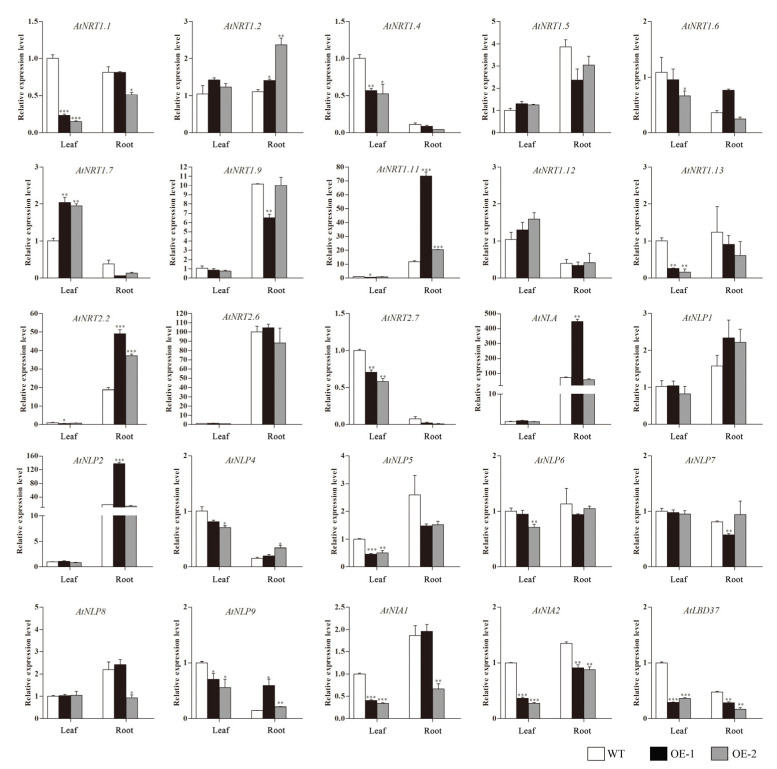
The expression levels of nitrate response related genes in WT and transgenic *Arabidopsis* plants hosting *CsLBD39* gene. The data are expressed as mean ± standard deviation of three biological replicates (*n* = 3). *AtSAND* was used as reference gene. Asterisks (*) indicate that the value is significant difference compared to the WT (* *p* < 0.05; ** *p* < 0.01; *** *p* < 0.001).

The effect of overexpression of *CsLBD39* gene on the expression of nitrate transport-related genes was analyzed. As showed in Figure 7, the expression levels of several nitrate transport genes, such as *AtNRT1.1*, *AtNRT1.4*, *AtNRT1.6*, *AtNRT1.11*, *AtNRT1.13*, *AtNRT2.7*, *AtNIA1*, and *AtNIA2* were significantly lower in transgenic *Arabidopsis* leaves than in the WT. Similarly, several TFs, such as *AtNLP5*, *AtNLP6*, *AtNLP9*, and *AtLBD37*, also showed a downward trend.

The expression levels of nitrate transport genes *AtNRT1.1*, *AtNRT1.4*, *AtNIA2*, *AtNRT1.7* and nitrate response TFs *AtNLP5*, *AtLBD37* in transgenic *Arabidopsis* roots were lower than WT. The expression levels of nitrate transport related genes *AtNRT1.11*, *AtNRT2.2* and nitrate response TFs *AtNLP2*, *AtNLP4* and *AtNLP9* in transgenic *Arabidopsis* roots were significantly higher than WT. These results suggested that overexpression of *CsLBD39* gene leads to changes in the expression of nitrate responsive genes in *Arabidopsis* plants.

### 2.8. Analysis of Nitrate, Anthocyanins and Chlorophyll Contents in Transgenic Arabidopsis Overexpressing CsLBD39 under Nitrate Treatment

**Figure 8:**
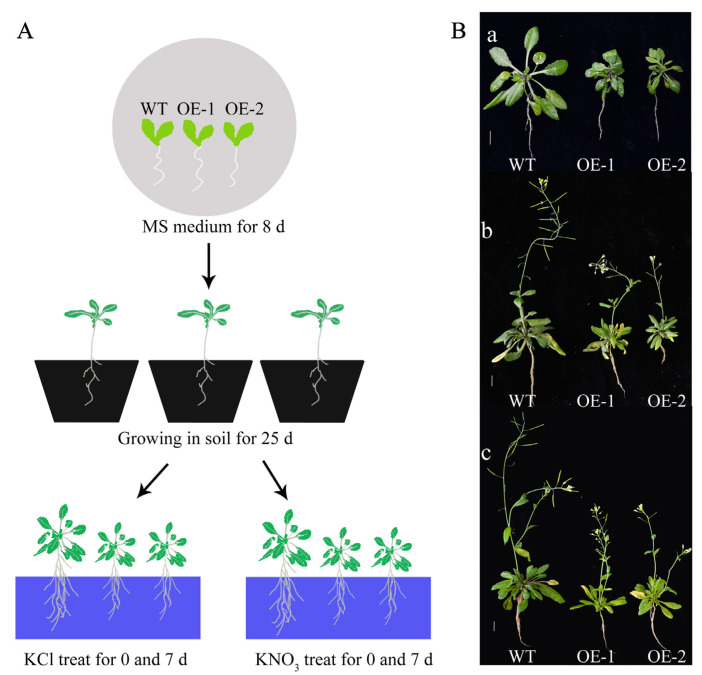
Effects of KCl and KNO_3_ treatments on growth of WT and transgenic *Arabidopsis* plants hosting *CsLBD39* gene. (**A**) Graphical abstract of the growth conditions of *Arabidopsis*. (**B**) The phenotypes of WT and transgenic *Arabidopsis*, (**a**) treats for 0 d, (**b**) KCl treatment for seven days, (**c**) KNO_3_ treatment for seven days, Bar = 1 cm.

**Figure 9:**
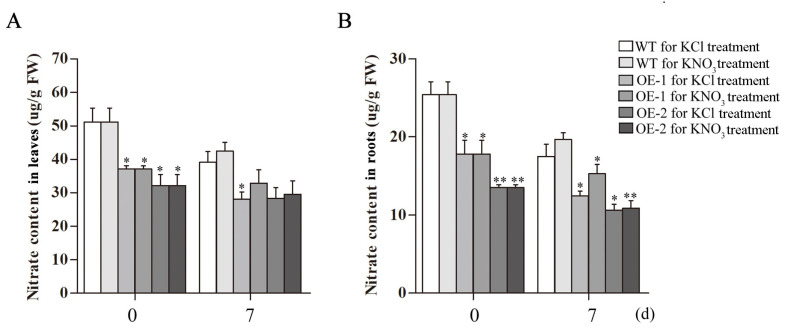
Analysis of nitrate contents in WT and transgenic *Arabidopsis* plants hosting *CsLBD39* gene under KCl and KNO_3_ conditions. (**A**) The nitrate contents in leaves. (**B**) The nitrate contents in roots. The data are expressed as mean ± standard deviation of three biological replicates (*n* = 3). Asterisks (*) indicate that the value is significant difference compared to the WT (* *p* < 0.05; ** *p* < 0.01).

**Figure 10:**
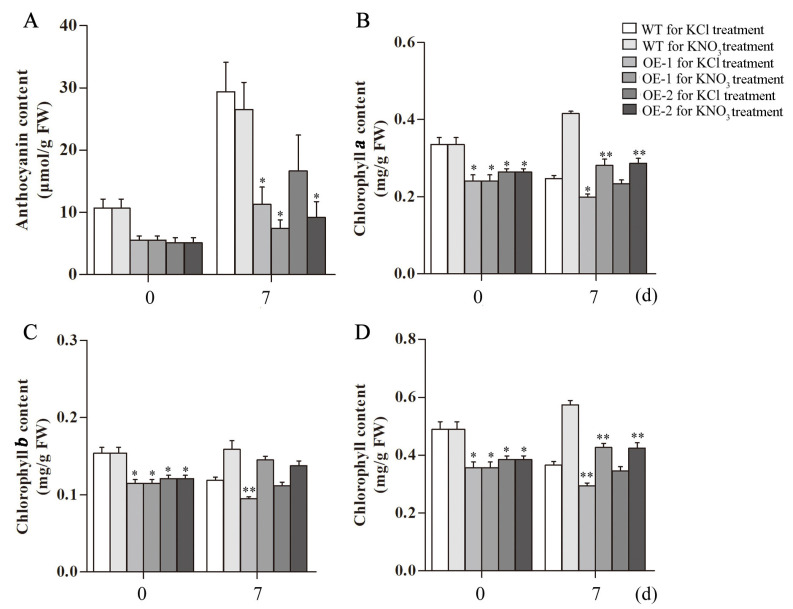
Analysis of anthocyanins and chlorophyll contents in WT and transgenic *Arabidopsis* plants hosting *CsLBD39* gene under KCl and KNO_3_ conditions. (**A**) The total anthocyanins contents of leaves in WT and transgenic *Arabidopsis* under KCl and KNO_3_ conditions. (**B**) The contents of chlorophyll *a* (**C**) chlorophyll *b*, and (**D**) total chlorophyll of leaves in transgenic and WT *Arabidopsis* under KCl and KNO_3_ conditions. The data are expressed as mean ± standard deviation of three biological replicates (*n* = 3). Asterisks (*) indicate that the value is significant difference compared to the WT (* *p* < 0.05; ** *p* < 0.01).

A detailed summary of *Arabidopsis* growth and treatment conditions is shown in Figure 8A, the WT and transgenic *Arabidopsis* were grown in the cultivation medium for 25 d and then transferred to KCl and KNO_3_ hydroponic nutrient solution for seven days (Figure 8B). The nitrate content in the leaves of the transgenic lines decreased after treatment, and the nitrate content under the KNO_3_ treatment was higher than that under the KCl treatment at seven days (Figure 9A). The same trend was observed in roots. The nitrate content in the roots of the transgenic plants was significantly reduced after treatment for seven days, the nitrate content in *Arabidopsis* roots under the KNO_3_ treatment was higher than that under the KCl treatment (Figure 9B). Nitrogen deficiency in plants will cause stress responses, which will affect the synthesis of anthocyanins. The anthocyanins content of *Arabidopsis* increased at seven days of KCl treatment, and the anthocyanins accumulation of transgenic lines was lower than that of WT. After seven days of KNO_3_ treatment, anthocyanins content in *Arabidopsis* plants increased compared with 0 d of KNO_3_ treatment, and transgenic lines also showed lower anthocyanins accumulation in contrast to WT. The anthocyanins content of *Arabidopsis* plants treated with KNO_3_ was still lower than that treated with KCl (Figure 10A).

The *Arabidopsis* plants in KCl treatment group showed more yellow leaves compared to that in KNO_3_ treatment group. The chlorophyll contents were measured. The results showed that the contents of chlorophyll *a* and chlorophyll *b* in the transgenic lines were significantly lower than those in the WT plants at 0 d of the KCl or KNO_3_ treatment. At seven days, both chlorophyll *a* and chlorophyll *b* of WT and transgenic *Arabidopsis* treated with KNO_3_ were higher than those treated with KCl, especially the chlorophyll *a* is significantly increased (Figure 10B–D).

### 2.9. Cytological Observation on Leaves and Stems of Transgenic Arabidopsis

**Figure 11:**
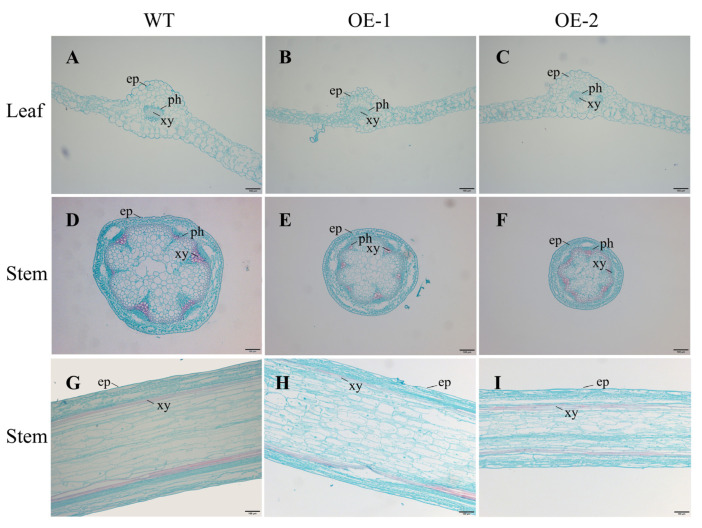
Histochemical staining sections of leaves and stems from WT and transgenic *Arabidopsis* plants hosting *CsLBD39* gene under KCl treatment. (**A**–**C**) Histochemical staining of leaves from WT and transgenic *Arabidopsis* plants hosting *CsLBD39* gene. (**D**–**F**) Histochemical staining of stem cross sections from WT and transgenic *Arabidopsis* plants hosting *CsLBD39* gene. (**G**–**I**) Histochemical staining of stem longitudinal sections from WT and transgenic *Arabidopsis* plants hosting *CsLBD39* gene. ep, epidermis; ph, phloem; xy, xylem. Scale bar = 100 μm.

**Figure 12:**
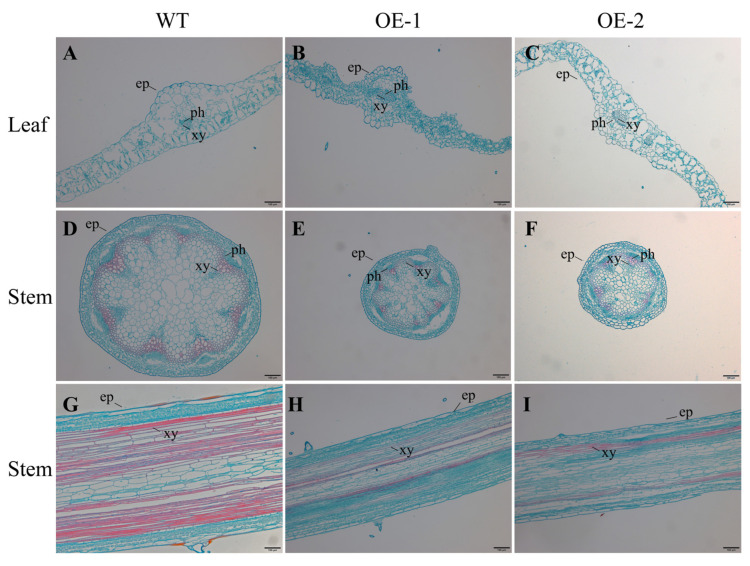
Histochemical staining sections of leaves and stems from WT and transgenic *Arabidopsis* plants hosting *CsLBD39* gene under KNO_3_ treat condition. (**A**–**C**) Histochemical staining of leaves from WT and transgenic *Arabidopsis* plants hosting *CsLBD39* gene. (**D**–**F**) Histochemical staining of stem cross sections from WT and transgenic *Arabidopsis* plants hosting *CsLBD39* gene. (**G**–**I**) Histochemical staining of stem longitudinal sections from WT and transgenic *Arabidopsis* plants hosting *CsLBD39* gene. ep, epidermis; ph, phloem; xy, xylem. Scale bar = 100 μm.

The transgenic *Arabidopsis* plants overexpressing the *CsLBD39* gene showed dwarfing and small rosette leaves in this study. The cytological morphological changes were further observed and analyzed. Leaves and stems of WT and transgenic *Arabidopsis* treated with KCl were selected for observation. The results showed that the phloem and xylem tissues of transgenic *Arabidopsis* leaves were smaller than that of WT (Figure 11). This phenomenon was also observed in the stem cell section of transgenic *Arabidopsis*. The diameter of stem cells was shortened and the cells became significantly smaller. The longitudinal observations of the stem showed that the cells in the transgenic *Arabidopsis* stem were small and compact (Figure 11). Under KNO_3_ treatment, the results of cell sections were similar to those of under KCl treatment (Figure 12). Regardless of the leaves or stems, the cells of transgenic *Arabidopsis* are reduced and compact, and the diameter of the stem cross section was also smaller. The cells of stem longitudinal section become slender and denser.

### 2.10. The Expression Analysis of Nitrate Uptake and Transport-Related Genes in Transgenic Arabidopsis Plants Overexpressing CsLBD39 under Nitrate Treatment

**Figure 13:**
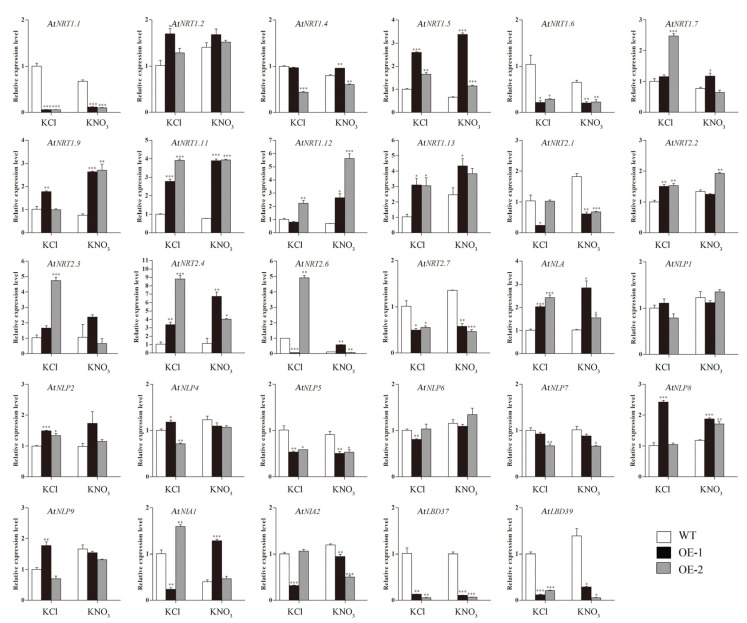
The expression levels of nitrate response related genes in leaves of WT and transgenic *Arabidopsis* plants hosting *CsLBD39* gene under KCl and KNO_3_ conditions. The data are expressed as mean ± standard deviation of three biological replicates (*n* = 3). Asterisks (*) indicate that the value is significant difference compared to the WT (* *p* < 0.05; ** *p* < 0.01; *** *p* < 0.001).

*Arabidopsis* plants were cultured in KCl and KNO_3_ hydroponic nutrient solution for seven days and sampled for RT-qPCR experiments. As is shown in Figure 13, the expression levels of *AtNRT1.1*, *AtNRT1.6*, *AtNRT2.1*, *AtNRT2.7*, *AtNLP5*, *AtNLP7*, *AtNIA2*, *AtLBD37*, and *AtLBD39* in transgenic lines were significantly lower than those in WT plants both under KCl treatment and KNO_3_ treatment. The expression levels of *AtNRT1.2*, *AtNRT1.5*, *AtNRT1.9*, *AtNRT1.11*, *AtNRT1.13*, *AtNRT2.4*, *AtNLA*, *AtNLP2*, and *AtNLP8* genes were significantly higher in transgenic lines than those in WT plants both under KCl treatment and KNO_3_ treatment.

**Figure 14:**
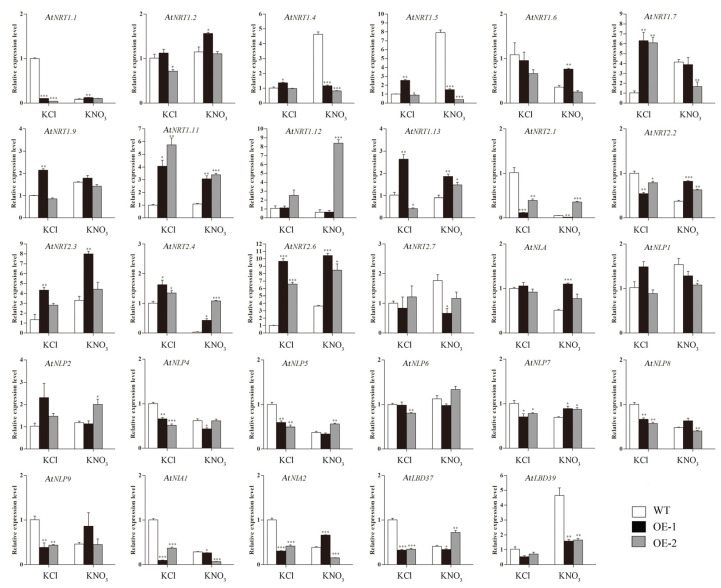
The expression levels of nitrate response related genes in roots of WT and transgenic *Arabidopsis* plants hosting *CsLBD39* gene under KCl and KNO_3_ conditions. The data are expressed as mean ± standard deviation of three biological replicates (*n* = 3). Asterisks (*) indicate that the value is significant difference compared to the WT (* *p* < 0.05; ** *p* < 0.01; *** *p* < 0.001).

The expression levels of genes related to nitrate response in roots were different under KCl and KNO_3_ treatments. As is shown in Figure 14, the expression levels of *AtNRT1.1*, *AtNRT2.1*, *AtNRT2.2*, *AtNLP5*, *AtNLP7*, *AtNLP8*, and *AtNLP9* in *Arabidopsis* roots of KCl treatment group were different from that of KNO_3_ treatment group, that is, the expression levels of these genes in transgenic lines were lower than those in WT plants under KCl treatment, whereas the results were opposite under KNO_3_ treatment. The expression levels of *AtNRT1.4*, *AtNRT1.5*, *AtNRT1.7*, and *AtNRT2.7* were decreased in transgenic lines than in WT under KNO_3_ treatment, and the opposite results were found under KCl treatment.

## 3. Discussion

As one of the main nutrients required by plants, nitrogen regulates many aspects of plant growth, development and metabolism. In some higher plants, inorganic nitrogen is mainly composed of two forms, NO_3_^−^ and NH_4_^+^, and nitrate is the preferential nitrogen source for most higher plants [28,29]. In tea plants, the absorption rate of ammonium nitrogen is higher than that of nitrate nitrogen [25]. The excessive application of ammonium nitrogen will cause soil acidification. Therefore, the research on the absorption and utilization of nitrate is also particularly important. The remobilization of nitrate between different organs is mainly mediated by nitrate transporters (NRTs) [30,31,32]. Previous studies have reported that overexpression of the *AtLBD* TF genes suppressed the expression of *NRT* and *NR* genes, thus controlling N utilization in *Arabidopsis* [11]. However, the LBD TFs that regulate nitrate-responsive genes in tea plants have not been studied so far. Searching for LBD TFs that regulate nitrate uptake and assimilation in tea plants is helpful for future molecular breeding in relevant fields.

LBD TFs play significant roles in plant growth, development, and metabolism [11,33,34]. Based on previous studies, LBD is classified as class I and class II [13]. In this work, sequence analysis showed that CsLBD39 belonged to class II subfamily of the LBDs and was homologous to AtLBD39 in *Arabidopsis*. In *Arabidopsis*, the expression of class II LBD genes, *LBD37*/*LBD38*/*LBD39*, are induced by nitrogen or glutamine [11]. Overexpression of *LBD37*/*LBD38*/*LBD39* genes inhibited the expression of *NRT* and *NR* genes, and changed the contents of nitrogen, nitrate and amino acids [11]. In this study, the expression of *CsLBD39* was induced by nitrate, the nitrate content was reduced, and the expression of *NRT* genes related to nitrate transport were inhibited in transgenic plants overexpressing *CsLBD39*. A similar phenomenon was found in apples, overexpression of *MdLBD13* altered the nitrate content and the expression of genes related to N metabolism in apple and *Arabidopsis* [16]. Studies have shown that AtLBD16, AtLBD29, and AtLBD18 regulate the formation of lateral roots [35,36]. The plant weight and root length of *Arabidopsis* overexpressing *CsLBD39* gene were changed under different KNO_3_ treatments. Overexpressing *CsLBD39* gene in *Arabidopsis* altered the root morphology under KNO_3_ treatment. These results suggested that CsLBD39 may act as a regulator to modulate the growth and development of plants under KNO_3_ treatment.

Yordanov and Busov proposed a mechanism model for the regulation of LBD in secondary woody growth, that is, *PtaLBD1* and *PtaLBD4* are expressed at the cambium/phloem boundary, could regulate secondary phloem development by inhibiting the expression of *ARBORKNOX1* and *ARBORKNOX2* genes, and could activate *APL* and other genes transcription to promote phloem development [37,38]. In *Eucalyptus grandis*, overexpression of *EgLBD37* gene resulted in some changes in the phenotype of the transgenic plants, namely, the plant became taller, the leaves became larger, the length of the internodes increased, the diameter of the stem increased, the total width of the cortical area and the xylem components of the secondary xylem increased significantly [39]. In contrast, the most pronounced phenotype of the *EgLBD29* transgenic plants was that all transgenic lines exhibited smaller plant height, reduced internode length and declined leaf size [39]. Similar reports have been found in this study, overexpression of the *CsLBD39* gene in *Arabidopsis* resulted in smaller and dwarf plants. Changes in plant phenotypes can cause cytological changes [40]. Further observation and analysis of cytological morphological changes in leaves and stems of transgenic *Arabidopsis* overexpressing *CsLBD39* gene found that the diameter of transgenic *Arabidopsis* stems was shortened and the cells in leaf and stem sections were smaller. These results suggested that CsLBD39 can affect plant growth and development.

## 4. Materials and Methods

### 4.1. Plant Materials, Growth Conditions

Tea plant cultivar ‘Longjing 43’ and wild type *Arabidopsis* ‘Columbia’ were selected as materials. ‘Longjing 43’ was planted in artificial climate room of the State Key Laboratory of Crop Genetics and Germplasm Enhancement of Nanjing Agricultural University. The condition of artificial climate room was 25/18 °C and 16/8 h of light/dark, with 70% relative humidity. The growing medium of tea plants is a mixture of peat, vermiculite and perlite (3:2:1; *v*/*v*). *Arabidopsis* plants was grown in the illumination incubator with the environment of 22/18 °C and 14/10 h of light/dark, as well as 70% relative humidity. The growing medium is a mixture of nutrient soil, vermiculite and perlite (18:6:1; *v*/*v*).

The young leaves (YL), mature leaves (ML), old leaves (OL), stems, flowers and roots of healthy tea plant with semblable physiological conditions were collected to analyze the expression of *CsLBD39* gene. One-year-old tea plant cuttings were transferred into a total nutrient solution as described by Zhang et al. [26]. The tea plants were cultivated for six weeks of normal N supply (2 mM). Subsequently, the tea plants were placed in a culture medium (without N, as CK) for 10 days, and then transferred to different KNO_3_ treatments with 0, 0.1, 1, and 10 mM. The tea roots treated with different KNO_3_ concentrations as mentioned above were collected after 2 h, frozen in liquid nitrogen, and stored at −80 °C for RT-qPCR tests. All samples were set up for three biological replicates.

### 4.2. RNA Extraction and cDNA Synthesis

The total RNA of tea plant and *Arabidopsis* samples were extracted using RNA extraction kit (Huayueyang, China; Pudi, China), and then the total RNA was reverse transcribed into cDNA using the HiScript II Q RT SuperMix for qPCR kit (Vazyme, Nanjing, China).

### 4.3. Isolation and Bioinformatics Analysis of CsLBD39

The sequence of CsLBD39 was downloaded from Tea Plant Information Archive (TPIA) (http://tpia.teaplant.org/index.html) (accessed on 17 January 2020) database [41]. The gene was cloned from ‘Longjing 43’ by a pair of primers (forward: 5′-ATGAGTTGCAATGGATGTCG-3′ and reverse: 5′-TCAGGTGAACAAGTTTAGAAG-3′) through polymerase chain reaction (PCR). The PCR product was first linked to the pMD19-T vector and then sequenced. Homologous LBD protein sequences and others were obtained using NCBI (https://www.ncbi.nlm.nih.gov/) (accessed on 2 April 2020) and Plant TFDB (http://planttfdb.gao-lab.org/index.php) (accessed on 2 April 2020). The MUSCLE program of MEGA 5 was used to carry out multiple alignments of protein sequences, and then phylogenetic trees were generated by the Neighbor-Joining method [42].

### 4.4. Subcellular Localization of CsLBD39

To confirm subcellular localization of CsLBD39, a pair of specific primers (forward: 5′-CACCATCACCATCACGCCATGATGAGTTGCAATGGATGTCG-3′ and reverse: 5′-CACTAGTACGTCGACCATGGCGGTGAACAAGTTTAGAAG-3′) was used to clone *CsLBD39* without stop codon. The PCR product was inserted into pA7 vector via *Nco* I site. Subsequently, the fusion construct (*35S:CsLBD39-GFP*) was generated. The *35S:CsLBD39-GFP* plasmid and the pA7 plasmid were separately bombarded into the onion epidermal cells (PDS-1000, Bio-Rad, Hercules, CA, USA) and then placed on MS medium in the dark condition [43]. After 14 h, the GFP expression signals was observed using a confocal laser scanning microscope (Zeiss, Germany) and photographed.

### 4.5. Transcriptional Activation Activity Analysis of CsLBD39

To verify the transcriptional activation activity of CsLBD39, a pair of specific primers (forward: 5′-ATGGCCATGGAGGCCGAATTCATGAGTTGCAATGGATGTCG-3′ and reverse: 5′-ATGCGGCCGCTGCAGGTCGACTCAGGTGAACAAGTTTAGAAG-3′) were used to clone *CsLBD39*. The PCR product was insert into the pGBKT7 vector via *Eco*R I and *Sal* I sites to generate a recombinant construct (pGBKT7-CsLBD39). Subsequently, the empty vector (pGBKT7, as the negative control), pCL1 plasmid (as the positive control), and pGBKT7-CsLBD39 were transformed into yeast strain Y2H, respectively. The yeast strains transformed with pCL1 plasmid was cultured on SD/Leu^−^ medium, while the yeast strains hosing pGBKT7-CsLBD39 or pGBKT7 were cultured on SD/Trp^−^ medium, respectively. After 3 d, positive clones were selected and inoculated on SD/His^−^Ade^−^ medium containing X-α-gal to examine whether they turned blue.

### 4.6. Overexpression Plasmid Construction and Transformation

The full length *CsLBD39* ORF was cloned using a pair of specific primers (forward: 5′-TTTACAATTACCATGGGATCCATGAGTTGCAATGGATGTCG-3′ and reverse: 5′-ACCGATGATACGAACGAGCTCTCAGGTGAACAAGTTTAGAAG-3′) and insert into the *Sac* I and *Bam*H I sites of pCAMBIA1301 vector that containing the β-glucosidase (*GUS*) gene to construct the recombinant plasmid pCAMBIA1301-CsLBD39. The expression of *CsLBD39* and *GUS* genes was driven by the 35S promoter, respectively. Simply put, the recombinant plasmid pCAMBIA1301-CsLBD39 was introduced into *Agrobacterium tumefaciens* strain GV3101. The *Arabidopsis* was transformed by *A*. *tumefaciens*-mediated genetic transformation using flower dipping method [44]. Transgenic *Arabidopsis* was screened on 1/2 MS medium containing hygromycin and carbenicillin. The transgenic lines were verified by GUS staining and PCR amplification tests.

### 4.7. Nitrate Treatment Conditions in Arabidopsis

WT and transgenic *Arabidopsis* seeds were plated on MS solid medium. The MS plate was placed in an illumination incubator for cultivation. *Arabidopsis* seedlings grown in MS medium for seven days were transferred to the cultivation medium. One month later, part of the plants was transferred to nutrient solution containing 1 mM KNO_3_ for seven days, and the other part was transferred to nitrogen free nutrient solution for seven days, KCl was used to control the difference in K^+^ concentration. *Arabidopsis* leaves after treatment were collected for RT-qPCR assay, anthocyanins, chlorophyll and nitrate contents determination. *Arabidopsis* roots were collected for RT-qPCR assay and nitrate contents determination.

MS nitrogen-free medium was purchased from PhytoTech LABS [45]. KNO_3_ was used as the sole nitrogen source. The final concentrations of adding KNO_3_ in MS nitrogen-free medium were 0.2 mM, 1 mM, and 5 mM. KCl with final concentrations of 4.8 mM, 4 mM, and 0 mM was added to MS nitrogen-free medium to supplement the corresponding concentration of K^+^. The seeds of WT and transgenic *Arabidopsis* were placed on the above-mentioned MS medium to evaluate the effects of KNO_3_ treatments at different concentrations on *Arabidopsis* plant fresh weight and root length.

### 4.8. Measurement of the Nitrate Content

WT and transgenic *Arabidopsis* were planted in a mixed substrate. 35-day-old *Arabidopsis* leaves and roots were collected for determination of nitrate content.

Briefly, 0.2 g of freeze-dried sample was added with deionized water and the mixture was boiled and centrifuged. The obtained supernatant was transferred into a new centrifuge tube, and salicylic acid-sulfuric acid solution was first added to mix, and then NaOH solution was added to react, cooling the reaction liquid to room temperature. The absorbance of reaction mixture was measured using microplate reader (Spectramax ID5) at 410 nm [1]. Three replicates were conducted.

### 4.9. Determination of Chlorophyll

WT and transgenic *Arabidopsis* were planted in a mixed substrate. 35-day-old *Arabidopsis* leaves were collected for determination of chlorophyll content.

The extraction and determination of chlorophyll (Chl) were carried out with reference to previous studies [46]. Briefly, the leaves are cut into pieces, 0.1 g fresh leaves added with 10 mL of the mixed extract (95% acetone: ethanol: distilled water = 4.5:4.5:1) and soaked in the dark for 24 h until the leaves turn completely white. The mixed extract was used as a blank control, the absorbance was measured by Spectramax ID5 at 645 nm and 663 nm, respectively. Three replicates were conducted.

### 4.10. Determination of Anthocyanins

WT and transgenic *Arabidopsis* were planted in a mixed substrate and grown to 35 d of age, and leaves were collected for determination of anthocyanins content.

The total content of anthocyanins in *Arabidopsis* leaves was determined by methanol-HCl method, as described in previous studies [47]. The absorbance was measured using Spectramax ID5 at 530, 620, and 650 nm. The relative anthocyanins concentration was calculated according to the formula. Each sample contains three independent biological replicates.

### 4.11. Histochemical Staining

Cytological observation was conducted according to the method described by Han with slightly modification [48,49]. The samples of leaves and stems are fixed and dehydrated, and then cut into slices with ultramicrotome (Leica, Weztlar, German). Generated slices were treated with multiple steps, including stained with safranin-*O*, washed with water, discolored with alcohol, and quick-dyed with green dye. Pictures was shot using a charge coupled device (CCD) camera.

### 4.12. Gene Expression Analysis

*CsGAPDH* and *CsTBP* were selected as reference genes [41,50], to explore the expression pattern of *CsLBD39* gene in different tissues and nitrate response. The expression levels of nitrate-responsive genes in WT and transgenic *Arabidopsis* were also analyzed. *AtSAND* and *AtActin2* were used as reference genes. RT-qPCR primers were consulted to previous studies and listed in Supplementary Table S1 [1,10,11,16,51,52]. RT-qPCR test was performed with 20 μL reaction mixtures using Hieff qPCR SYBR Green Master Mix (Yeasen, Shanghai, China) on CFX96 system (Bio-Rad, Hercules, CA, USA). The relative expressions of genes were calculated using the 2^−ΔΔCT^ method. Three separate biological replicates were set.

### 4.13. Statistical Analysis

Data were analyzed by SPSS 17.0 software. The difference significance of gene expression levels in tea plant were detected by Duncan’s multiple-range test at a 0.05 probability. The statistical differences of data between WT and transgenic *Arabidopsis* were analyzed by one-way analysis of variance and indicated by asterisks (*) (* *p* < 0.05; ** *p* < 0.01; *** *p* < 0.001).

## 5. Conclusions

In conclusion, a novel transcription factor, named as CsLBD39, was identified from ‘Longjing 43’. CsLBD39 is an LBD Class II transcription factor. Subcellular localization, transcriptional activation, and overexpression in *Arabidopsis* were performed to confirm its function. Overexpression of CsLBD39 decreased the nitrate content and the expression of nitrate transport-related genes in transgenic *Arabidopsis* plants. These results provided evidence that CsLBD39 may play a negative regulatory factor in the nitrate response pathway of tea plants.

## Data Availability

In this section, transcriptional data, physiological and anatomic metabolic data were measured by the authors themselves.

## Supplementary Materials

The following supporting information can be downloaded at: https://www.mdpi.com/article/10.3390/ijms23169294/s1.

## References

[1] Xu N., Wang R., Zhao L., Zhang C., Wang Y. (2016). The *Arabidopsis* NRG2 protein mediates nitrate signaling and interacts with and regulates key nitrate regulators. Plant Cell.

[2] Liu Z.W., Li H., Liu J.X., Wang Y., Zhuang J. (2020). Integrative transcriptome, proteome, and microRNA analysis reveals the effects of nitrogen sufficiency and deficiency conditions on theanine metabolism in the tea plant (*Camellia sinensis*). Hortic. Res..

[3] Crawford N.M. (1995). Nitrate: Nutrient and signal for plant growth. Plant Cell.

[4] Ho C.H., Lin S.H., Hu H.C., Tsay Y.F. (2009). CHL1 Functions as a Nitrate Sensor in Plants. Cell.

[5] Walch-Liu P., Forde B.G. (2008). Nitrate signalling mediated by the NRT1.1 nitrate transporter antagonises L-glutamate-induced changes in root architecture. Plant J..

[6] Stitt M. (1999). Nitrate regulation of metabolism and growth. Curr. Opin. Plant Biol..

[7] Shuai B., Reynaga-Pena C.G., Springer P.S. (2002). The lateral organ boundaries gene defines a novel, plant-specific gene family. Plant Physiol..

[8] Bi Y.M., Wang R.L., Zhu T., Rothstein S.J. (2007). Global transcription profiling reveals differential responses to chronic nitrogen stress and putative nitrogen regulatory components in *Arabidopsis*. BMC Genom..

[9] Wang R., Xing X., Crawford N. (2007). Nitrite acts as a transcriptome signal at micromolar concentrations in *Arabidopsis* roots. Plant Physiol..

[10] Yu L.H., Wu J., Tang H., Yuan Y., Wang S.M., Wang Y.P., Zhu Q.S., Li S.G., Xiang C.B. (2016). Overexpression of *Arabidopsis* NLP7 improves plant growth under both nitrogen-limiting and -sufficient conditions by enhancing nitrogen and carbon assimilation. Sci. Rep..

[11] Rubin G., Tohge T., Matsuda F., Saito K., Scheible W.R. (2009). Members of the LBD family of transcription factors repress anthocyanin synthesis and affect additional nitrogen responses in *Arabidopsis*. Plant Cell.

[12] Majer C., Hochholdinger F. (2011). Defining the boundaries: Structure and function of LOB domain proteins. Trends Plant Sci..

[13] Matsumura Y., Iwakawa H., Machida Y., Machida C. (2009). Characterization of genes in the ASYMMETRIC LEAVES2/LATERAL ORGAN BOUNDARIES (AS2/LOB) family in *Arabidopsis thaliana*, and functional and molecular comparisons between AS2 and other family members. Plant J..

[14] Wang X., Zhang S., Su L., Liu X., Hao Y. (2013). A genome-wide analysis of the LBD (LATERAL ORGAN BOUNDARIES Domain) gene family in *Malus domestica* with a functional characterization of MdLBD11. PLoS ONE.

[15] Husbands A., Bell E.M., Shuai B., Smith H.M.S., Springer P.S. (2007). LATERAL ORGAN BOUNDARIES defines a new family of DNA-binding transcription factors and can interact with specific bHLH proteins. Nucleic Acids Res..

[16] Li H.H., Liu X., An J.P., Hao Y.J., Wang X.F., You C.X. (2017). Cloning and elucidation of the functional role of apple MdLBD13 in anthocyanin biosynthesis and nitrate assimilation. Plant Cell Tissue Org..

[17] Albinsky D., Kusano M., Higuchi M., Hayashi N., Kobayashi M., Fukushima A., Mori M., Ichikawa T., Matsui K., Kuroda H. (2010). Metabolomic screening applied to rice FOX *Arabidopsis* lines leads to the identification of a gene-changing nitrogen metabolism. Mol. Plant.

[18] Konishi M., Yanagisawa S. (2013). Arabidopsis NIN-like transcription factors have a central role in nitrate signalling. Nat. Commun..

[19] Lee H.W., Kim M.J., Kim N.Y., Lee S.H., Kim J. (2013). LBD18 acts as a transcriptional activator that directly binds to the EXPANSIN14 promoter in promoting lateral root emergence of *Arabidopsis*. Plant J..

[20] Lee H.W., Kim J. (2013). EXPANSINA17 up-regulated by LBD18/ASL20 promotes lateral root formation during the auxin response. Plant Cell Physiol..

[21] Xia E.H., Zhang H.B., Sheng J., Li K., Zhang Q.J., Kim C., Zhang Y., Liu Y., Zhu T., Li W. (2017). The tea tree genome provides insights into tea flavor and independent evolution of caffeine biosynthesis. Mol. Plant.

[22] Wang X., Feng H., Chang Y., Ma C., Wang L., Hao X., Li A., Cheng H., Wang L., Cui P. (2020). Population sequencing enhances understanding of tea plant evolution. Nat. Commun..

[23] Li H., Liu Z.W., Wu Z.J., Wang Y.X., Teng R.M., Zhuang J. (2018). Differentially expressed protein and gene analysis revealed the effects of temperature on changes in ascorbic acid metabolism in harvested tea leaves. Hortic. Res..

[24] Wang Y.X., Liu Z.W., Wu Z.J., Li H., Wang W.L., Cui X., Zhuang J. (2018). Genome-wide identification and expression analysis of GRAS family transcription factors in tea plant (*Camellia sinensis*). Sci. Rep..

[25] Yang Y.Y., Li X.H., Ratcliffe R.G., Ruan J.Y. (2012). Characterization of ammonium and nitrate uptake and assimilation in roots of tea plants. Russ. J. Plant Physiol..

[26] Zhang F., Wang L., Bai P., Wei K., Cheng H. (2020). Identification of regulatory networks and hub genes controlling nitrogen uptake in tea plants [*Camellia sinensis* (L.) O. Kuntze. J. Agric. Food Chem..

[27] Lin C.C., Tsai Y.S., Lin Y.S., Chiu T.Y., Hsiung C.C., Lee M.I., Simpson J.C., Hsu C.N. (2007). Boosting multiclass learning with repeating codes and weak detectors for protein subcellular localization. Bioinformatics.

[28] Miller A.J., Cramer M. (2005). Root nitrogen acquisition and assimilation. Plant Soil.

[29] Poitout A., Crabos A., Petřík I., Novák O., Krouk G., Lacombe B.T., Ruffel S. (2018). Responses to systemic nitrogen signaling in *Arabidopsis* roots involve trans-zeatin in shoots. Plant Cell.

[30] Hirel B., Bertin P., Quillere I., Bourdoncle W., Attagnant C., Dellay C., Gouy A., Cadiou S., Retailliau C., Falque M. (2001). Towards a better understanding of the genetic and physiological basis for nitrogen use efficiency in maize. Plant Physiol..

[31] Jukanti A.K., Fischer A.M. (2008). A high-grain protein content locus on barley (*Hordeum vulgare*) chromosome 6 is associated with increased flag leaf proteolysis and nitrogen remobilization. Physiol Plantarum..

[32] Liu W., Sun Q., Wang K., Du Q., Li W.X. (2017). Nitrogen limitation adaptation (NLA) is involved in source-to-sink remobilization of nitrate by mediating the degradation of NRT1.7 in *Arabidopsis*. New Phytol..

[33] Bortiri E., Chuck G., Vollbrecht E., Rocheford T., Martienssen R., Hake S. (2006). ramosa2 encodes a LATERAL ORGAN BOUNDARY domain protein that determines the fate of stem cells in branch meristems of maize. Plant Cell.

[34] Chalfun-Junior A., Franken J., Mes J.J., Marsch-Martinez N., Pereira A., Angenent G.C. (2005). ASYMMETRIC LEAVES2-LIKE1 gene a member of the AS2/LOB family, controls proximal-distal patterning in *Arabidopsis* petals. Plant Mol. Biol..

[35] Lee H.W., Cho C., Pandey S.K., Park Y., Kim M.J., Kim J. (2019). LBD16 and LBD18 acting downstream of ARF7 and ARF19 are involved in adventitious root formation in *Arabidopsis*. BMC Plant Biol..

[36] Feng Z., Sun X., Wang G., Liu H., Zhu J. (2012). LBD29 regulates the cell cycle progression in response to auxin during lateral root formation in *Arabidopsis thaliana*. Ann. Bot..

[37] Yordanov Y.S., Busov V. (2011). Boundary genes in regulation and evolution of secondary growth. Plant Signal. Behav..

[38] Yordanov Y.S., Regan S., Busov V. (2010). Members of the LATERAL ORGAN BOUNDARIES DOMAIN transcription factor family are involved in the regulation of secondary growth in Populus. Plant Cell.

[39] Lu Q., Shao F., Macmillan C., Wilson I.W., van der Merwe K., Hussey S.G., Myburg A.A., Dong X., Qiu D. (2018). Genomewide analysis of the lateral organ boundaries domain gene family in *Eucalyptus grandis* reveals members that differentially impact secondary growth. Plant Biotechnol. J..

[40] Zhou J., Lee C., Zhong R., Ye Z.H. (2009). MYB58 and MYB63 are transcriptional activators of the lignin biosynthetic pathway during secondary cell wall formation in Arabidopsis. Plant Cell.

[41] Wei C., Yang H., Wang S., Zhao J., Liu C., Gao L., Xia E., Lu Y., Tai Y., She G. (2018). Draft genome sequence of Camellia sinensis var. sinensis provides insights into the evolution of the tea genome and tea quality. Proc. Natl. Acad. Sci. USA.

[42] Tamura K., Peterson D., Stecher G., Peterson N., Kumar S., Nei M. (2011). MEGA5: Molecular evolutionary genetics analysis using maximum likelihood, evolutionary distance, and maximum parsimony methods. Mol. Biol. Evol..

[43] Liu J.X., Feng K., Duan A.Q., Li H., Yang Q.Q., Xu Z.S., Xiong A.S. (2019). Isolation, purification and characterization of an ascorbate peroxidase from celery and overexpression of the *AgAPX1* gene enhanced ascorbate content and drought tolerance in *Arabidopsis*. BMC Plant Biol..

[44] Li T., Huang Y., Khadr A., Wang Y.-H., Xu Z.-S., Xiong A.-S. (2020). DcDREB1A, a DREB-binding transcription factor from *Daucus carota*, enhances drought tolerance in transgenic *Arabidopsis thaliana* and modulates lignin levels by regulating lignin-biosynthesis-related genes. Environ. Exp. Bot..

[45] Murashige T., Skoog F. (1962). A revised medium for rapid growth and bio assays with tobacco tissue cultures. Physiol. Plantarum..

[46] Yu X., Hu S., He C., Zhou J., Qu F., Ai Z., Chen Y., Ni D. (2019). Chlorophyll metabolism in postharvest tea (*Camellia sinensis* L.) leaves: Variations in color values, chlorophyll derivatives, and gene expression levels under different withering treatments. J Agric. Food Chem..

[47] Ren Y.R., Zhao Q., Yang Y.Y., Zhang T.E., Wang X.F., You C.X., Hao Y.J. (2021). The apple 14-3-3 protein MdGRF11 interacts with the BTB protein MdBT2 to regulate nitrate deficiency-induced anthocyanin accumulation. Hortic. Res..

[48] Han M.H., Yang N., Wan Q.W., Teng R.M., Duan A.Q., Wang Y.H., Zhuang J. (2021). Exogenous melatonin positively regulates lignin biosynthesis in *Camellia sinensis*. Int. J. Biol. Macromol..

[49] Liu J.X., Jiang Q., Tao J.P., Feng K., Li T., Duan A.Q., Wang H., Xu Z.S., Liu H., Xiong A.S. (2021). Integrative genome, transcriptome, microRNA, and degradome analysis of water dropwort (*Oenanthe javanica*) in response to water stress. Hortic. Res..

[50] Wu Z.J., Tian C., Jiang Q., Li X.H., Zhuang J. (2016). Selection of suitable reference genes for qRT-PCR normalization during leaf development and hormonal stimuli in tea plant (*Camellia sinensis*). Sci. Rep..

[51] Lu K., Li T., Jian H., Wei C., Rui Z., Liu M., Yu M., Fan Y., Ma J., Wei S. (2018). qPrimerDB: A thermodynamics-based gene-specific qPCR primer database for 147 organisms. Nucleic Acids Res..

[52] Menz J., Li Z., Schulze W.X., Ludewig U. (2016). Early nitrogen-deprivation responses in *Arabidopsis* roots reveal distinct differences on transcriptome and (phospho-) proteome levels between nitrate and ammonium nutrition. Plant J..

