# Supplementary material for: Isolation and Characterization of an LBD Transcription Factor CsLBD39 from Tea Plant (Camellia sinensis) and Its Roles in Modulating Nitrate Content by Regulating Nitrate-Metabolism-Related Genes
Source: Int J Mol Sci. 2022 Aug 18;23(16):9294. doi: 10.3390/ijms23169294 (PMC9409460; doi:10.3390/ijms23169294)
Supplement: Supplementary file 1 [file ijms-23-09294-s001.zip › Supplementary Figure S1.pdf]

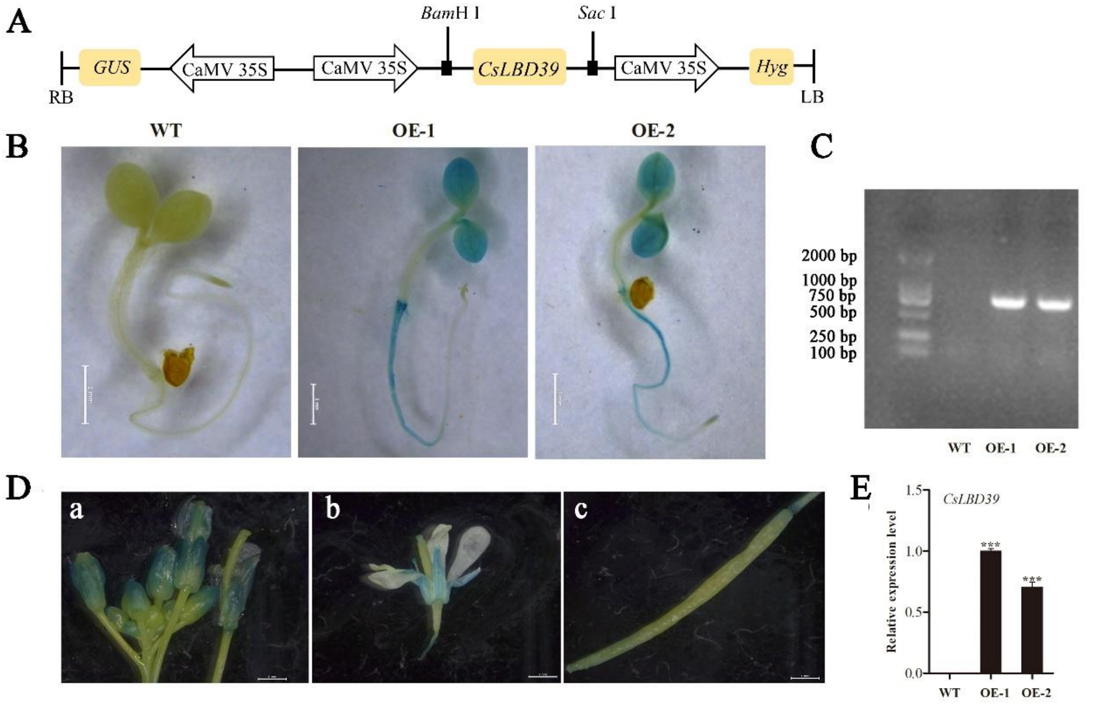

Supplementary Figure S1: GUS staining and PCR amplification of *Arabidopsis* transformed with a *CsLBD39* gene from tea plant. (A) Diagram of the *CsLBD39* overexpression construct. (B) Histochemical GUS assays of WT and transgenic *Arabidopsis*. (C) PCR amplification of *CsLBD39* from cDNA of WT and transgenic *Arabidopsis*. (D) Histochemical staining of transgenic *Arabidopsis* hosting *CsLBD39*, (a) inflorescence, (b) flower, (c) silique. (E) The expression levels of *CsLBD39* in WT and transgenic *Arabidopsis*. *AtActin2* was used as reference gene. Scale bars = 1 mm. The data are expressed as mean ± standard deviation of three biological replicates (n=3). Asterisks (*) indicate that the value is significant difference compared to the WT (* $P < 0.05$; ** $P < 0.01$; *** $P < 0.001$).
