# Supplementary material for: Isolation and Characterization of an LBD Transcription Factor CsLBD39 from Tea Plant (Camellia sinensis) and Its Roles in Modulating Nitrate Content by Regulating Nitrate-Metabolism-Related Genes
Source: Int J Mol Sci. 2022 Aug 18;23(16):9294. doi: 10.3390/ijms23169294 (PMC9409460; doi:10.3390/ijms23169294)
Supplement: Supplementary file 1 [file ijms-23-09294-s001.zip › Supplementary Table S1.pdf]

Supplemental Table 1. Primers used for RT-qPCR in this study

Gene	Forward primer (5'-3')	Reverse primer (5'-3')	References
CsLBD39	CTTGACGCCATCATCATCGG	TGTAAAGTCGTCGTCCCAGATT	-
CsGAPDH	TTGGCATCGTTGAGGGTCT	CAGTGGGAACACGGAAAGC	[41]
CsTBP	GGCGGATCAAGTGTTGGAAGGGAG	ACGCTTGGGATTGTATTCGGCATTA	[50]
AtSAND	AACTCTATGCAGCATTTGATCCACT	TATTGCATATCTTTATCGCCATC	[52]
AtActin2	TGTGCCAATCTACGAGGGTTT	TTTCCCGCTCTGCTGTTGT	-
AtNRT1.1	CTGCCACACACTGAACAATTCC	CCCGCTTCCTGATCCCTTAT	[11]
AtNRT2.2	GGAAAGATTCTATGGTACGCCG	CGGCGATAACATTGTCTGTGC	[11]
AtNIA1	GGCTACGCTTATTCTGGAGGAGGT	TGGTGGTCAAGCTCACAAACACTC	[11]
AtNIA2	GCCGACGAAGAAGGTTGGTGGTAT	GAAGAATCTCCTCGTGACATGGCG	[11]
AtLBD37	TGCTTTGTTTCAGTCGTTGCTC	TGCTCCGTTAACTGGATTGACA	[11]
AtLBD39	GAACTCCAACGTCCTGCTTTGT	ATACCAACCGCTCCGTTAACC	[11]
AtNRT1.4	GACCGTGCCATAATGCCTTT	TGAACTAGATTTCGCAACGGA	[1]
AtNRT1.7	CAACAGTCAGTTTCCAGAGCACAT	CGACAGTCACAAGGAAACTACTAAGGTA	[1]
AtNRT1.6	CAATTCAAGTTTCTGGACAAAGCTG	GACCTCTTCAACTTCTTGGATGCTA	[1]
AtNRT2.6	TTGTTTCCCGCCGTTCTCTTG	TCTGTCCATTCCGCTCCATAG	[1]
AtNRT2.4	CGTTATGGCTGTGCTTTCCTC	ACCTCACCGTTATGTACCCTCC	-
AtNRT2.1	AGTCGCTTGCACGTTACCTG	ACCCTCTGACTTGGCGTTCTC	[16]
AtNRT1.2	GTATTTGGCGAATGCGAGTAAT	TTGAAAAGTGGAGAAGAAAGCG	[51]
AtNRT1.5	ATCTACTTGATCGCATTGGGAT	ATCCGAGGTTTAAAGCAAGGTA	[51]
AtNRT1.9	TTTCAACATGAAGAGCATCACG	TCCTATTTCCTTAGCGCATTGA	[51]
AtNRT1.11	GGCATTGATCAAAGTCATACCC	GTAACTGAAACGAACTTTGGCT	[51]
AtNRT2.7	TTGTTTTCCGGTGATGATGTTC	GACGTAGACTACCACTAACCAC	[51]
AtNRT1.12	GTAAAACCATCACCGTGTGTAG	ATCGATATTAATGCGAATGCGG	[51]
AtNRT1.13	AAGAAGGTTGGTTAGGCGATAA	TTTCGCCATTTTCTAAAGCCTC	[51]
AtNRT2.3	ACTAAAACCGACATTGGAAACG	GAACATAACGCTGGTCCAATAC	[51]
AtNLP1	TCAAGGAGCCTCTTGTTCTTG	ATGTCGCTTTCACTTTGATGG	[10]
AtNLP2	TGGTACTGGCACTTCCTTCA	TGATGGTGGTGACTTTGGAG	[10]
AtNLP4	TCACAACAAGCCAAGACCAC	GGGTTTCACTGGAGCAACAT	[10]
AtNLP5	ATTCACCACCTGCAAAGTCC	TATCCGTGGGATCTTCCTTG	[10]
AtNLP6	CTCACCAGAGTCGGTAAGCA	CGGAGACCCATTCTCTATCG	[10]
AtNLP7	GAGTTTGCCCGACGACAATGAAG	GGCCTCCATCAGTACCTTGAACAG	[10]
AtNLP8	CAGCGAGAGTGGATCAACAA	CAACTGATGGCTCGAACTTG	[10]
AtNLP9	AATCGAGCGAGGTAATGGAA	GAACTTCCCAGCAAAACTGC	[10]
AtNLA	GAGCGACTCTGTTTTGTTGATC	CATGTACTCTTCATACTTCTTACA	[10]
